# ASNET: A Novel AI Framework for Accurate Ankylosing Spondylitis Diagnosis from MRI

**DOI:** 10.3390/biomedicines11092441

**Published:** 2023-09-01

**Authors:** Nevsun Pihtili Tas, Oguz Kaya, Gulay Macin, Burak Tasci, Sengul Dogan, Turker Tuncer

**Affiliations:** 1 Department of Physical Medicine and Rehabilitation, Health Sciences University Elazig Fethi Sekin City Hospital, Elazig 23280, Turkey; nevsunpihtili@gmail.com; 2 Department of Orthopedics and Traumatology, Elazig Fethi Sekin City Hospital, Elazig 23280, Turkey; oguzkayamd@gmail.com; 3 Department of Radiology, Beyhekim Training and Research Hospital, Konya 42060, Turkey; gulaymacin@gmail.com; 4 Vocational School of Technical Sciences, Firat University, Elazig 23119, Turkey; btasci@firat.edu.tr; 5 Department of Digital Forensics Engineering, College of Technology, Firat University, Elazig 23119, Turkey

**Keywords:** ASNet, ankylosing spondylitis, deep feature engineering, biomedical image classification, information fusion

## Abstract

Background: Ankylosing spondylitis (AS) is a chronic, painful, progressive disease usually seen in the spine. Traditional diagnostic methods have limitations in detecting the early stages of AS. The early diagnosis of AS can improve patients’ quality of life. This study aims to diagnose AS with a pre-trained hybrid model using magnetic resonance imaging (MRI). Materials and Methods: In this research, we collected a new MRI dataset comprising three cases. Furthermore, we introduced a novel deep feature engineering model. Within this model, we utilized three renowned pretrained convolutional neural networks (CNNs): DenseNet201, ResNet50, and ShuffleNet. Through these pretrained CNNs, deep features were generated using the transfer learning approach. For each pretrained network, two feature vectors were generated from an MRI. Three feature selectors were employed during the feature selection phase, amplifying the number of features from 6 to 18 (calculated as 6 × 3). The k-nearest neighbors (kNN) classifier was utilized in the classification phase to determine classification results. During the information phase, the iterative majority voting (IMV) algorithm was applied to secure voted results, and our model selected the output with the highest classification accuracy. In this manner, we have introduced a self-organized deep feature engineering model. Results: We have applied the presented model to the collected dataset. The proposed method yielded 99.80%, 99.60%, 100%, and 99.80% results for accuracy, recall, precision, and F1-score for the collected axial images dataset. The collected coronal image dataset yielded 99.45%, 99.20%, 99.70%, and 99.45% results for accuracy, recall, precision, and F1-score, respectively. As for contrast-enhanced images, accuracy of 95.62%, recall of 80.72%, precision of 94.24%, and an F1-score of 86.96% were attained. Conclusions: Based on the results, the proposed method for classifying AS disease has demonstrated successful outcomes using MRI. The model has been tested on three cases, and its consistently high classification performance across all cases underscores the model’s general robustness. Furthermore, the ability to diagnose AS disease using only axial images, without the need for contrast-enhanced MRI, represents a significant advancement in both healthcare and economic terms.

## 1. Introduction

Ankylosing spondylitis is a common inflammatory rheumatic disease that affects the axial skeleton, causing characteristic inflammatory lower back pain [1]. The disease originates from the sacroiliac joints and extends to involve the spine, causing inflammatory pain. Both genetic and external factors affect how the disease develops [2]. The most common clinical symptom is back pain. However, the diagnosis is usually skipped, and there are delays of an average of 8–11 years from the start of the complaint [3]. Patients may receive unnecessary treatments or experience delayed recovery due to misdiagnosis. This can lead to disabilities that burden society [4]. AS is typically diagnosed using the modified New York criteria, which consider both clinical symptoms and imaging findings. Conventional radiography is employed to identify structural changes in the sacroiliac joint, which are crucial for confirming the diagnosis. Conventional X-rays are the primary method for diagnosis. However, X-rays cannot detect the early stages of the disease in which inflammation occurs, and irreversible structural changes have not yet occurred [5]. AS is less common compared to cases of lumbar disc herniation and degeneration. Diagnosing AS in institutions with specialists is quick and easy with direct radiographs. However, in primary healthcare facilities, there may not be experts capable of detecting AS early, which can delay diagnosis [6]. Spinal and extra-spinal involvement, as well as delayed diagnosis, can lead to limited functionality. Patients may become unable to perform their daily tasks. The limitation in functionality is essential due to the socio-economic, psychosocial, and financial consequences it may cause for the individual and society.

In addition, X-rays cannot detect the early stages of the disease, where inflammation occurs and irreversible structural changes have not yet happened. The early stages of the disease can be diagnosed through magnetic resonance imaging (MRI) [7,8,9]. MRI can detect early sacroiliitis symptoms such as subchondral bone marrow edema. MRI is a crucial diagnostic tool in managing spondyloarthritis (SpA) as it can detect active and chronic changes before structural changes occur, with the ability to show active lesions and structural changes [10]. Imaging is crucial in evaluating patients with inflammatory back pain in SpA, as it helps in early detection and appropriate treatment to prevent irreversible changes [11]. Treating bone marrow edema involves addressing sacroiliac and spinal edema, which can halt disease progression and avoid disability. Early diagnosis and personalized treatment are essential for the successful management of AS [12]. Other inflammatory spine lesions, such as sacroiliitis, spondylitis, and spondylodiscitis, can be visualized in the early stages with MRI. Early diagnosis can be established, and the likelihood of disability caused by the disease can be reduced. Providing necessary care to patients can prevent the burden of the disease on society [13].

Artificial intelligence (AI) has significant potential in detecting AS, especially when used with MRI [12,14]. AI algorithms can be trained on large datasets to learn AS-specific patterns and utilize this information for analyzing new MR images. AS-specific findings in MR images include inflammation and erosion in the sacroiliac joints, bridging of spine bones, and spinal joint abnormalities [15]. AI algorithms can identify these abnormalities, analyze the intensity and distribution of various features in these images, and assess the likelihood of AS. When combined with patients’ symptoms, clinical evaluations, and other medical data, AI also has the potential to improve the accuracy of AS diagnosis. The analysis of symptoms and risk factors enables AI algorithms to provide important assessments for AS diagnosis. This way, early detection of AS can be achieved, and time can be saved to initiate appropriate treatment [9]. However, AI-based AS detection should be supported by the supervision and verification of a doctor. AI systems are designed to inform and enhance the diagnostic process for doctors but cannot entirely replace them. Considering the complexity of AS and other factors influencing diagnosis, the ultimate decision always rests with a healthcare professional [16].

### 1.1. Literature Review

In the literature, there are different studies on disease diagnosis using MR images [17,18,19,20,21]. Among these studies, those on AS are presented as follows. Han et al. [22] developed an automated algorithm for quantifying and grading AS–hip arthritis utilizing MRI. The algorithm incorporates deep learning-based segmentation and classification networks to accurately identify inflammatory regions and assess the severity of AS in MRIs. The algorithm’s performance was validated through a retrospective analysis involving 141 cases, divided into a derivation cohort (101 patients) and a validation cohort (40 patients). The results revealed median percentages of bone marrow edema (BME) for each grade, with grade 1 (<15%) at 36%, grade 2 (15–30%) at 42%, and grade 3 (≥30%) at 22% within the derivation group. The algorithm achieved an impressive accuracy rate of 85.7% in accurately diagnosing AS through examining 835 MRIs. It made correct decisions for 31 out of 40 AS test cases. Navarani et al. [23] conducted a study to assess the effectiveness of various cardiovascular risks in patients diagnosed with AS. The study compared traditional cardiovascular markers with ML approaches. The data obtained from 133 AS patients determined that 18 had cardiovascular events. The algorithms displayed limited discriminative ability, except for RRS and SCORE. C-reactive protein (CRP) emerged as the most critical variable for assessing cardiovascular risk in AS. The ML algorithms SVM, RF, and Knn achieved AUC values of 70.00%, 73.00%, and 64.00%, respectively. Feature analysis highlighted the significance of CRP, while systolic blood pressure (SBP) and hypertension treatment were deemed less important. Li et al. [12] presented an AI-based tool for diagnosing and treating AS. The ensemble deep learning model achieved high performance values in an external test set. It attained a precision of 90.00%, a recall of 89.00%, and an AUC of 96.00%. Zunti et al. [24] conducted a study using medical imaging techniques to detect erosion, an early symptom of AS. They utilized both statistical machine learning and deep learning algorithms to analyze computed tomography (CT) images, factoring in the patient’s age. The random forest classifiers demonstrated outstanding performance, achieving an accuracy of 96%, a recall of 93%, and an area under the receiver operator characteristic curve (ROC AUC) of 97.00% for erosion detection. Lin et al. [25] used a deep learning algorithm that utilizes the short tau inversion recovery (STIR) sequence in MRI to detect active inflammatory sacroiliitis. To train the algorithm, they used original MRIs and “fake-color” images generated from ground truth masks outlining bone marrow edema, which indicates inflammation. The study involved 326 participants diagnosed with axial spondyloarthritis (SpA) and 63 with non-specific back pain. The results showed that the algorithm exhibited comparable sensitivity and specificity to a radiologist’s interpretation and outperformed a rheumatologist’s assessment. This suggests the algorithm’s potential for diagnosing SpA and evaluating disease activity. Notably, the algorithms successfully identified inflammatory sacroiliitis in 1398 MR images from 228 participants, with 3944 MRI scans from 161 participants showing no inflammation. The algorithms trained on the original and fake-color image datasets demonstrated mean sensitivities of 92.00% and 90.00% and mean specificities of 92.00% and 93.00%, respectively. Bressem et al. [26] used an artificial neural network (ANN) to detect sacroiliitis in axial spondyloarthritis (axSpA) using conventional radiographs of the sacroiliac joints. They utilized a total of 2011 radiographs in their study. The neural network demonstrated excellent performance, achieving areas under the ROC curve (AUCs) of 97.00% and 94.00% for the validation and test datasets, respectively. The sensitivity and specificity of the neural network for the validation set were 88.00% and 95.00%, respectively. In comparison, for the test set, the sensitivity was 92.00%, and the specificity was 81.00%. Shenkman et al. [27] employed a supervised machine learning technique using an automatic algorithm to detect and grade sacroiliitis in computerized tomography scans. Based on 484 sacroiliac joints in their experimental results, they achieved a binary classification accuracy of 92.00% and a sensitivity of 95.00%. Furthermore, the algorithm achieved a three-class case classification accuracy of 86.00% and a sensitivity of 82.00%. Bressem et al. [28] conducted a study based on deep learning for the diagnosis of axial spondyloarthritis. Using their proposed method, they achieved 88% sensitivity and 78% specificity results.

### 1.2. Motivation

The primary motivation behind this study was to explore the potential for classification using our novel hybrid pre-trained model. Designed to be adaptable for various outcomes, this model inspired the development of a classification method named ASNet for assessment. To spotlight the efficacy of ASNet and showcase its classification prowess, we curated an image dataset composed of AS patients and control subjects. This dataset encompasses MRI scans from two distinct anatomical planes: coronal and axial, in addition to contrast-enhanced MRIs. A pivotal drive for our research was the ambition to diagnose AS using either axial or coronal MRIs, eliminating the need for contrast-enhanced MRI. While numerous studies on AS detection exist in the academic literature, there remains a conspicuous absence of a dedicated, publicly available dataset tailored for this objective. In response to this gap, we have released the MRI dataset we gathered to augment research visibility and inspire further exploration.

### 1.3. Contributions

This paper presents a comprehensive study on the detection and classification of AS disease using MRIs, with a special emphasis on harnessing the power of deep learning and advanced feature engineering methodologies. The following are the primary contributions we made:

- We collected a unique MRI dataset, catering specifically to AS diagnosis, which contains three distinct cases. This dataset serves as an invaluable resource for researchers aiming to improve AS disease diagnosis through computational means.
- Our study introduced a state-of-the-art deep feature engineering model that leverages three well-known pre-trained convolutional neural networks: DenseNet201, ResNet50, and ShuffleNet. We effectively generated profound and discerning features crucial for AS diagnosis through the transfer learning methodology.
- We implemented three distinct feature selectors, resulting in an expansion of our feature vectors. Expanding 6 to 18 (= 6 × 3) features enables the model to make more informed decisions, optimizing classification performance.
- Through employing the kNN classifier, we have demonstrated the high classification capability of the generated and selected features.
- Our model has generated 16 voted results in the information fusion through deploying the IMV algorithm. Our proposed deep feature engineering model is self-organized since this model selects the best outcome among the generated 34 (= 18 classifier-wise + 16 voted) outcomes.
- One of the most pivotal aspects of our work is the capability of our model to diagnose AS disease using only axial images, eliminating the need for contrast-enhanced MRI. This breakthrough has profound implications regarding reducing healthcare costs and increasing the accessibility of AS diagnosis.

## 2. Dataset

This study introduced a new dataset sourced from Elazığ Fethi Sekin City Hospital. In 2018, images were acquired using the Philips Multiva 1.5 Tesla MRI device manufactured in the Netherlands. Clinical records of patients from 2018 to 2023 were sourced from the hospital management system.

Patients who sought treatment at the rheumatology clinic and were radiologically diagnosed with ankylosing spondylitis had their axial, coronal, and contrast-enhanced MRI images incorporated into the AS study group. For comparison, healthy individuals without any medical conditions constituted the control group. Only AS patients satisfying the Assessment of SpondyloArthritis International Society criteria (2009) were considered for the study.

The axial dataset comprised images from 527 individuals: 260 with AS and 267 from the healthy control group. It contained 2110 MRIs (1000 AS and 1110 from healthy controls). Within the AS subset, axial images of 124 females and 136 males were included. The average age deduced from axial images for the AS group was 43.1 ± 1.30 years. For the healthy control group, the axial image dataset encompassed 125 females and 142 males, with an average age of 39.2 ± 4.25 years.

Contrast-enhanced MRIs were sourced from 821 participants: 152 with AS and 669 from the healthy control group. This group contained 1232 MRIs (223 AS and 1009 from healthy controls). Data from 78 females and 74 males were incorporated within the AS subset for contrast-enhanced MRIs, having an average age of 41.7 ± 5.21 years. In contrast, the healthy control group for these MRIs consisted of 345 females and 324 males, with an average age of 34.5 ± 2.32 years.

Coronal images featured 668 participants: 340 with AS and 328 from the healthy control group. This dataset held 2005 MRIs (1000 AS and 1005 from healthy controls). Within the AS subset for coronal images, 155 females and 185 males were considered, with an average age of 37.57 ± 3.45 years. The healthy control group, on the other hand, comprised 136 females and 212 males, having an average age of 35.25 ± 2.27 years.

**Figure 1:**
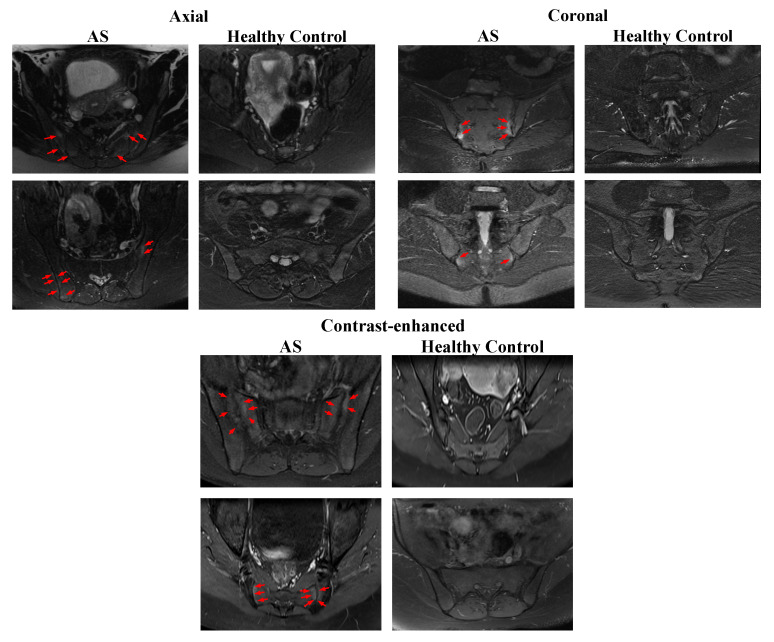
Sample images.

Participant details are documented in Table 1. Sample images sourced from the gathered dataset are depicted in Figure 1.

The axial and coronal short tau inversion recovery (STIR) MRI images show increased fluid signal due to bone marrow edema (red arrows) involving the sacral and iliac side adjacent to the sacroiliac joint. The coronal fat-saturated contrast-enhanced T1-weighted image shows enhancement at the sacroiliac joint bilateral (red arrows). (See Figure 1.)

## 3. The Proposed Method

**Figure 2:**
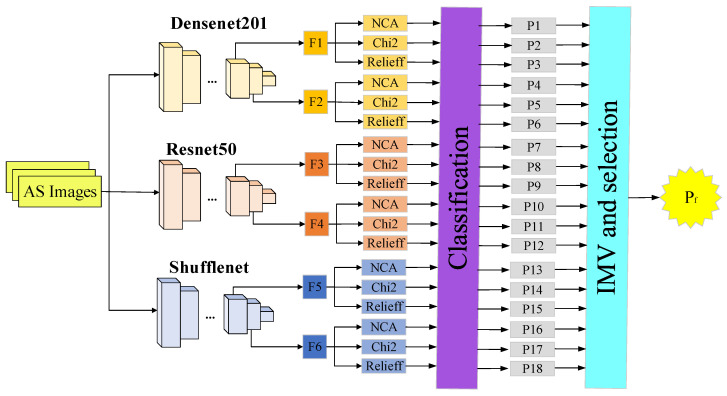
Block diagram of the proposed method.

In this study, we present a model called ASNet. ASNet is a deep feature engineering model that utilizes pre-trained DenseNet201 [29], ResNet50 [30], and ShuffleNet [31], convolutional neural networks (CNNs) which have been previously trained on the ImageNet1K dataset. ASNet generates six feature vectors (F_1_, F_2_…F_6_) through employing these three CNNs. To extract more features, we apply three feature selection methods: NCA [32], ReliefF [33], and Chi2 [34], resulting in a total of 18 (= 3 × 6) feature vectors (P_1_, P_2_…P_18_). Using the 18 obtained feature vectors, we employ a k-nearest neighbor (kNN) classifier to obtain prediction vectors. Finally, we aggregate the eighteen ASNet results using the iterative majority voting (IMV) algorithm. In this study, we combined the best feature vectors, feature selection method, and classifier to achieve high accuracy. For more detailed information about the ASNet model presented in this section, refer to Figure 2, where a schematic representation is provided.

### 3.1. Feature Extraction

ShuffleNet, ResNet-50, and DenseNet-201 are significant architectural examples in the field of deep learning. ShuffleNet is designed to achieve effective performance even on devices with low computational power, as it possesses a lightweight structure. Thanks to its original channel shuffling mechanism, ShuffleNet achieves high accuracy with fewer parameters and computations. On the contrary, ResNet-50 is a model that enables the training of deep networks through incorporating skip connections, also referred to as residual connections. It is commonly used in environments with moderate computational resources. DenseNet-201, on the other hand, has a deeper network structure characterized by dense connections. These dense connections enable better feature learning and a more efficient model with a lower parameter count.

*Step 1:* ShuffleNet, ResNet50, and DenseNet201 CNN models are used to extract features from the AS images. The fully connected classification layers used for feature extraction are as follows: F1 = fc1000, F2 = avg_pool, F3 = fc1000, F4 = avg_pool, F5 = node_200, F6 = node_202. Herein, we gave the names of the feature generation layers.

### 3.2. Feature Selection

Feature selection aims to eliminate irrelevant or less informative features from a dataset and determine the most appropriate subset of features that will enhance classification or clustering performance. NCA [32] evaluates the impact of each feature on classification performance through considering the neighborhood of data points using proximity information. Its goal is to identify the most suitable feature subset to maximize performance in tasks such as classification or clustering.

ReliefF [35] is a filtering method for feature selection that measures the impact of features on a classification task. Initially, it selects a random example among feature vectors and finds its nearest neighbors. The algorithm then computes the distinctions between neighbors of the same class and neighbors of different classes.

Chi2 [34] is a statistical test for feature selection and is effective when working with categorical features. This test determines whether there is a dependency relationship between two categorical variables. It evaluates features based on their relationships with the target variable and helps filter out insignificant features with high *p*-values.

*Step 2:* Various feature selection methods such as NCA, Chi2, and Relieff are employed. These feature selectors are used to choose the most informative 272 features.

### 3.3. Classification

In this article, the focus has been on classification using the k-nearest neighbors (KNN) algorithm. The performance and results of the algorithm have been examined through considering the hyperparameters of KNN. The hyperparameter ‘number of neighbors’ determines the number of nearest neighbors to be used for classification or prediction. In this study, the number of neighbors is chosen as 1, which means the label or value of a data point is predicted solely based on the information from its closest neighbor. The distance metric preferred for this purpose is the Euclidean distance metric, which calculates the distance between data points in the feature space using this metric. In the model, the ‘Distance weight’ hyperparameter is set uniformly, meaning all neighbors contribute equally to the prediction. The data has also been standardized to mitigate scale discrepancies between features.

*Step 3:* The selected features are used to train a kNN classifier with 10-fold cross-validation (CV) for the classification task.

*Step 4:* The kNN classifier generates 18 separate classification predictions (P1, P2,…, P18) on the test data after performing a 10-fold CV.

### 3.4. Information Fusion

Iterative majority voting (IMV) serves as an efficient method for amalgamating predictions from models predicated on multiple outputs in classification scenarios. This algorithm was postulated by Dogan et al. [36]. The paramount objective of IMV lies in augmenting the classification efficacy of the prediction vectors engendered by classifiers. To achieve this, IMV adopts an iterative framework. Initially, outcomes are organized in descending order based on their classification accuracies. These top-performing vectors are then integrated into the loop, with the IMV leveraging the mode function to yield voted outcomes. In this study, the loop’s range was between 3 and 18, producing 16 (= 18 − 3 + 1) voted outcomes.

*Step 5:* Apply IMV to the 18 generated classifier-wise outputs. In this model, the iteration range is from 3 to 18. Therefore, 16 voted outcomes have been created. Moreover, the mode function has been utilized as the majority voting function.

*Step 6:* Select the best output from the voted outputs per the classification accuracy.

## 4. Experimental Results

In this study, we introduced a new ASNet model. We utilized the MATLAB 2023a version for implementing the model. We downloaded pre-trained networks such as ShuffleNet, ResNet50, and DenseNet201 to train the model using the deep learning toolbox. Layer activation was used to extract features from the images. For feature selection, we employed the feature selection function and the classification learner toolbox in MATLAB to generate the kNN (k-nearest neighbors) code for classifiers. We encoded this process using iterative feature selection and the IMV functions. For our applications, we used a personal computer configured with 128 GB of memory, a 13th-generation Intel Core-i9 3.00 GHz processor, and the Windows 11 operating system. ASNet produced 18 separate results, and these 18 individual results were combined using the IMV algorithm to calculate the majority voting results (16 voted results). We tabulated the accuracy results for the collected axial, coronal, and contrast-enhanced AS MRIs in Table 2.

Table 2 lists the results garnered using the introduced ASNet method. Best performances are highlighted in bold. The minimum accuracy achieved for the gathered axial, coronal, and contrast-enhanced AS MRIs was 97.11%, 89.73%, and 90.34%, respectively. The most outstanding results are indicated in bold, with the combination of DenseNet201 equipped with a GAP layer, the NCA feature selector, and the kNN classifier standing out as the top-performing method. Specifically, with the best-performing method (Method 4), accuracy results of 99.65%, 99.10%, and 95.13% were secured for the axial, coronal, and contrast-enhanced AS MRIs in that order. These outcomes attest to the superior classification performance of the proposed ASNet method in comparison to other techniques. The results underscore the efficacy of the axial, coronal, and contrast-enhanced AS MRIs in the model, indicating that the proposed approach aptly processes these images.

**Figure 3:**
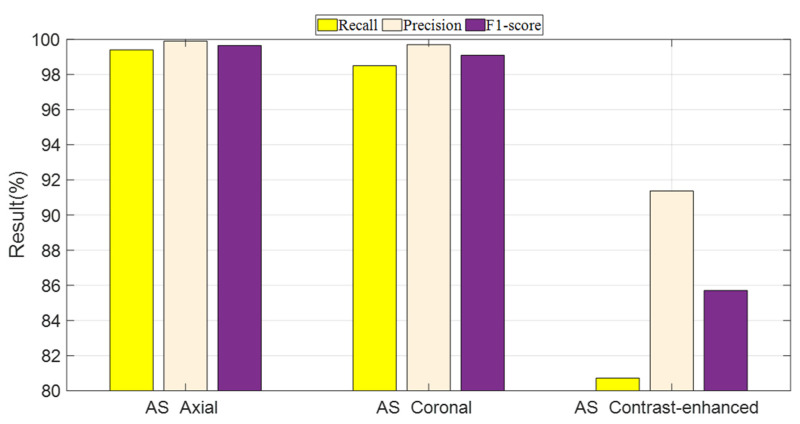
Performance metrics of the collected datasets.

Performance metrics, including recall, precision, and F1-score, were evaluated for Method 4 (DenseNet201 + avg_pool layer + NCA + kNN), deployed on the three datasets. These metrics are illustrated in Figure 3.

**Figure 4:**
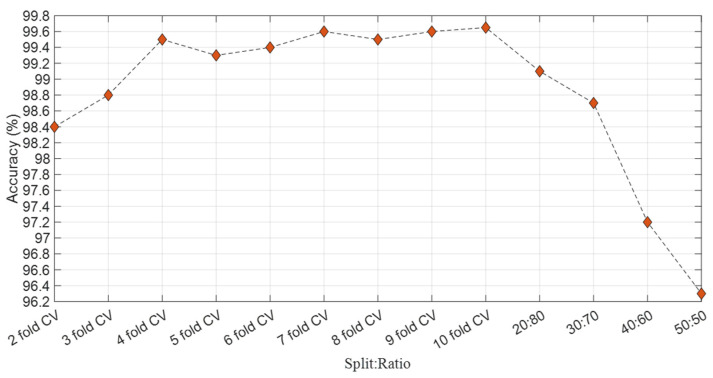
Accuracy results for different split ratios.

This study achieved the most superior/best accuracy using the 10-fold CV technique, commonly favored in the literature. As a result, we opted for the 10-fold CV method for our analysis. The accuracy results have been computed from the DenseNet201 + avg_pool layer + NCA + kNN combination with various validation techniques, illustrated in Figure 4.

**Figure 5:**
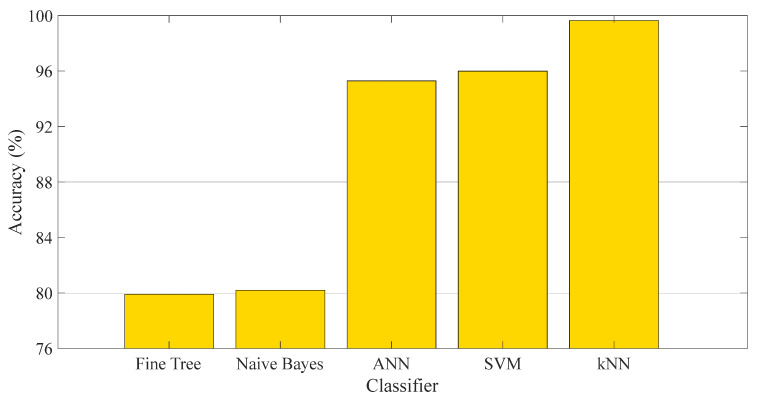
Accuracy results for different classifiers.

We chose the kNN classifier for our proposed method because it consistently delivered superior results compared to other classifiers. The accuracy results of various classifiers, when integrated with DenseNet201 + avg_pool layer + NCA + 10-fold CV for axial images, are illustrated in Figure 5. This test was conducted to underscore the supremacy of the kNN classifier.

**Figure 6:**
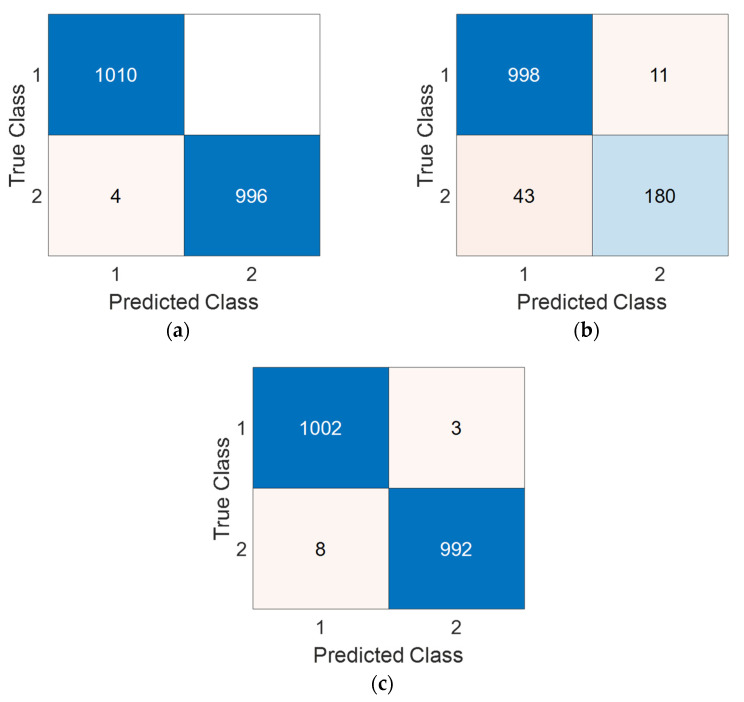
Confusion matrices of the proposed method for the used three cases: (**a**) axial, (**b**) contrast-enhanced, (**c**) coronal. Moreover, the classes have been depicted using numbers, and these are 1—healthy control, 2—AS.

ASNet generated 18 distinct outcomes during the classification phase, of which 16 were consolidated using IMV. The optimal outcomes are attributed to the outputs voted upon via the IMV process. To provide a comprehensive view, the confusion matrices of these final results of the ASNet per the used dataset are displayed in Figure 6.

We derived four performance metrics from the confusion matrices: accuracy, recall, precision, and F1-score through true negative, true positive, false positive, and false negative values. These computed performance metrics have been tabulated in Table 3.

The best performance results among the ASNet performance metrics were achieved with 99.80% accuracy on the axial image dataset. The second-best performance was obtained with 99.45% accuracy on the coronal image dataset. Lastly, an accuracy of 95.62% was achieved on the contrast-enhanced image dataset.

## 5. Discussion

AS is a chronic inflammatory rheumatic disease primarily known for causing inflammation in specific spinal joints and sacroiliac joints. MRI scans can diagnose and monitor the condition, which has become crucial for patient follow-up. The continuous progress of technology has enabled the application of artificial intelligence in analyzing medical imaging data, including MRIs. This study developed and assessed a novel approach, utilizing artificial intelligence to detect AS through analyzing axial, coronal, and medicated MRIs. The promising results highlight the potential of AI in improving AS diagnosis and patient care. The model generated deep instance features using pre-trained ShuffleNet, ResNet50, and DenseNet201 networks. Pre-trained ShuffleNet, ResNet50, and DenseNet201 models (trained on the ImageNet 1M dataset) were used to extract features from the original images, including layers F1 = fc1000, F2 = avg_pool, F3 = fc1000, F4 = avg_pool, F5 = node_200, and F6 = node_202. The extracted features were selected using the NCA, Chi2, and Relieff algorithms. The selected features were then classified using the kNN classifier, resulting in 18 prediction vectors. The IMV algorithm was applied to these prediction vectors, yielding excellent results. A comparative analysis of studies from the literature is delineated in Table 4.

Koo et al. [37] utilized a deep learning model to grade the corners of cervical and lumbar vertebral bodies in patients with AS. They employed digital radiographic images and developed a convolutional neural network model to classify the corners of the vertebral bodies. The average accuracy, sensitivity, and specificity values were 91.60%, 80.28%, and 94.24%, respectively. Zheng et al. [38] presented a deep learning-based model for hip bone marrow edema and synovitis in spondyloarthritis patients using MRI. They compared four deep learning models and found that U-Net achieved segmentation accuracy for femoral heads and inflammatory lesions. With U-Net, they achieved an accuracy of 88.48%. In contrast, our proposed model (ASNet) demonstrated a marked improvement, achieving over 95% accuracy on our dataset that includes axial, coronal, and contrast images, underscoring its superior performance.

**Figure 7:**
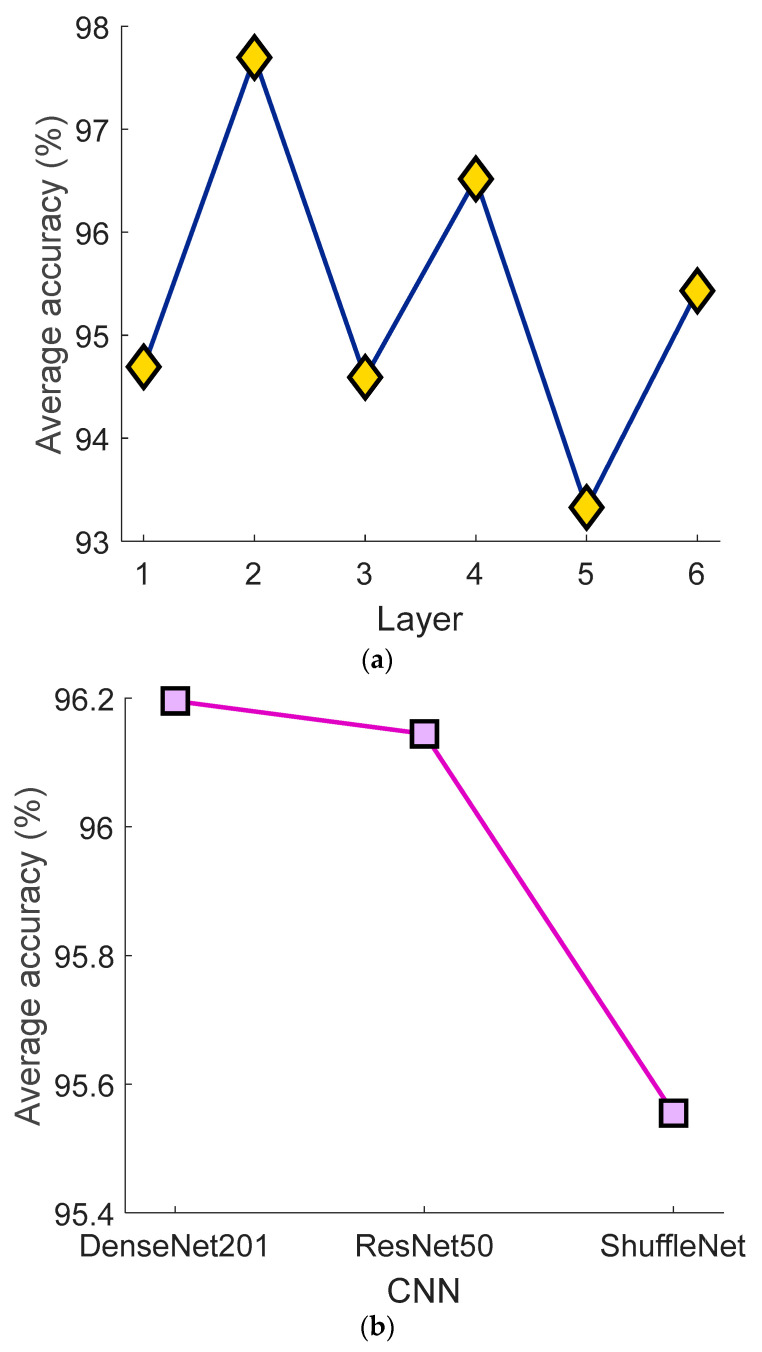
The average classification accuracies of the feature generation approximation of the proposed ASNet. (**a**) Average classification accuracies of the used layers. 1: fc100 layer of the DenseNet201, 2: avg_pool layer of the DenseNet201, 3: fc100 layer of the ResNet50, 4: avg_pool layer of the ResNet50, 5: Node200 layer of the ShuffleNet, 5: Node202 layer of the ShuffleNet. (**b**) Average classification accuracies of the used CNNs.

Additionally, we calculated the average classification accuracies of the employed feature generation models to demonstrate the effectiveness of each feature extraction methodology. Consequently, results from both the layers and CNNs were taken into account, and the average classification accuracies of these models are displayed below (Figure 7).

The findings, advantages, and limitations of this research are also discussed below.

*Findings:*

- MRI scans are crucial for diagnosing and monitoring AS, a chronic inflammatory rheumatic disease known for causing inflammation in specific spinal and sacroiliac joints.
- Artificial intelligence is progressively being applied to analyze medical imaging data, including MRIs.
- The proposed model utilizes artificial intelligence to detect AS through analyzing axial, coronal, and medicated MRIs.
- The model employs deep instance features using pre-trained ShuffleNet, ResNet50, and DenseNet201 networks.
- Feature extraction from original images is achieved using pre-trained ShuffleNet, ResNet50, and DenseNet201 models.
- Extracted features were selected using the NCA, Chi2, and Relieff algorithms.
- Using the kNN classifier, these features resulted in 18 prediction vectors.
- The IMV algorithm applied to these prediction vectors yields excellent results.
- The proposed ASNet outperformed other models, achieving an accuracy of over 95% on axial, coronal, and contrast images.

*Advantages:*

- The use of AI in this domain has the potential to greatly improve AS diagnosis and overall patient care.
- The proposed model combines the strengths of multiple pre-trained networks (ShuffleNet, ResNet50, and DenseNet201) for feature extraction.
- The model uses a multi-stage process, from feature extraction to selection and then classification, ensuring a robust mechanism for detection.
- The application of the IMV algorithm results in highly accurate predictions.
- The proposed model (ASNet) demonstrated superior performance compared to other models in the literature, underscoring its effectiveness.

*Limitations:*

- The study primarily discusses the results and advantages of the proposed model but does not explicitly mention its limitations. Based on the provided text, no explicit limitations of the proposed model are stated. Further insights or additional context might be required to list our potential limitations.

## 6. Conclusions

This study introduced a novel deep feature engineering model for the detection of AS. AI-based detection demonstrated high accuracy and sensitivity in diagnosing AS. Three cases were created from the original dataset, utilizing axial, coronal, and contrast-enhanced MRIs. Pre-trained ShuffleNet, ResNet50, and DenseNet201 networks were employed to extract deep features. The NCA, Chi2, and Relieff algorithms were used for feature selection. kNN and 10-fold CV were employed as classifiers. Finally, the IMV algorithm was utilized. The results obtained from axial MRIs were highly satisfactory, with the model correctly classifying almost all samples with a 99.80% accuracy rate. Moreover, the high sensitivity (100.00%) and F1-score (99.80%) values demonstrated that the model accurately detected positive cases while minimizing false positives. For coronal MRIs, accuracy, recall, precision, and F1-score were achieved at 99.45, 99.20, 99.70, and 99.45, respectively. For contrast-enhanced MRIs, accuracy, recall, precision, and F1-score were obtained at 95.62, 80.72, 94.24, and 86.96, respectively. The analyses performed on axial, coronal, and contrast-enhanced MRIs showed that artificial intelligence achieved a level of success comparable to clinical experts in detecting AS-related pathologies. This represents a significant advancement that could aid in early diagnosis and prompt the initiation of treatment in clinical applications.

To contribute to this field in the future, we will increase the number of images in the dataset we collect. In our model, three CNNs have been utilized for feature extraction. We will augment the number of CNNs used and employ iterative feature selectors. We can develop next-generation automatic AS detection applications through incorporating popular explainable artificial intelligence (XAI) models.

## Figures and Tables

**Table 1:** Information of participants.

	Male	Female	Total	Age (Mean ± SD)	MRI Images
**Axial AS**	136	124	260	43.1 ± 1.30	1000
**Axial Healthy Control**	142	125	267	39.2 ± 4.25	1110
**Coronal AS**	185	155	340	37.57 ± 3.45	1000
**Coronal Healthy Control**	212	136	348	35.25 ± 2.27	1005
**Contrast-enhanced AS**	74	78	152	41.7 ± 5.21	223
**Contrast-enhanced Healthy Control**	324	345	669	34.5 ± 2.32	1009

**Table 2:** The classifier-wise results of the proposed ASNet.

No	Generation Method				AS Axial Accuracy (%)	AS Coronal Accuracy (%)	AS Contrast- Enhanced Accuracy (%)
1	**DenseNet201**	fc1000 layer	NCA	kNN	98.81	94.16	92.61
2		fc1000 layer	Chi2	kNN	97.51	93.07	90.91
3		fc1000 layer	RF	kNN	98.71	94.66	91.80
4		avg_pool layer	NCA	kNN	**99.65**	**99.10**	**95.13**
5		avg_pool layer	Chi2	kNN	99.15	98.30	94.40
6		avg_pool layer	RF	kNN	99.60	98.90	95.05
7	**ResNet50**	fc1000 layer	NCA	kNN	98.41	94.66	93.10
8		fc1000 layer	Chi2	kNN	97.11	92.12	90.83
9		fc1000 layer	RF	kNN	98.56	94.16	92.37
10		avg_pool layer	NCA	kNN	99.15	98.50	93.18
11		avg_pool layer	Chi2	kNN	98.81	97.11	91.48
12		avg_pool layer	RF	kNN	99.10	98.00	93.34
13	**ShuffleNet**	Node200 layer	NCA	kNN	98.76	91.37	92.45
14		Node200 layer	Chi2	kNN	97.26	89.73	90.34
15		Node200 layer	RF	kNN	97.76	90.97	91.31
16		Node202 layer	NCA	kNN	98.71	95.61	92.78
17		Node202 layer	Chi2	kNN	98.76	94.81	91.48
18		Node202 layer	RF	kNN	98.66	96.36	91.72

**Table 3:** ASNet performance metric results.

Dataset	Class	Accuracy (%)	Recall (%)	Precision (%)	F1-Score (%)
Axial	Healthy Control	99.80	100.00	99.61	99.80
	AS		99.60	100.00	99.80
Coronal	Healthy Control	99.45	99.70	99.21	99.45
	AS		99.20	99.70	99.45
Contrast-enhanced	Healthy Control	95.62	98.91	95.87	97.37
	AS		80.72	94.24	86.96

**Table 4:** Comparative results.

Study	Method	Number of Samples	Split Ratio	The Results (%)
Koo et al. (2022) [37]	ResNet	5083 cervical, 5245 lumbar lateral	20:80	Accuracy: 91.60 Sensitivity: 80.28 Specificity: 94.24
Zheng et al. (2023) [38]	U-Net	1945 MRI	5-fold CV	Accuracy: 88.48
Our Proposed Model	ShuffleNet, ResNet50, DenseNet201, NCA, Chi2, Relieff, kNN, IMV	Axial 1000 AS 1110 healthy control Coronal 1000 AS 1005 healthy control Contrast-enhanced 223 AS 1009 healthy	10-fold CV	Axial Accuracy: 99.80 Recall: 99.60 Precision: 100 F1-Score: 99.80 Coronal Accuracy: 99.45 Recall: 99.20 Precision: 99.70 F1-Score: 99.45 Contrast-enhanced Accuracy: 95.62 Recall: 80.72 Precision: 94.24 F1-Score: 86.96

## Data Availability

Dataset is available upon request.
